# A Narrow Definition of Clinical Robustness

**DOI:** 10.2196/41715

**Published:** 2023-09-21

**Authors:** Jaclyn Marshall, Alexandra Yurkovic, Todd Thames, Ami Parekh

**Affiliations:** 1 Included Health San Francisco, CA United States

**Keywords:** digital health, research, virtual care

We read with great interest the article “Assessing the Clinical Robustness of Digital Health Startups: Cross-Sectional Observational Analysis” by Day et al [1]. We appreciate the authors highlighting the importance of assessing the quality and impact of the growing digital health industry. However, we believe the definition of “clinical robustness” was far too narrow and not equally applicable to the heterogeneous group of digital health startups in the study.

The authors applied a unique approach using publicly available data from ClinicalTrials.gov and Food and Drug Administration (FDA) filings to assess the clinical robustness of 224 digital health startups. While we acknowledge that defining clinical robustness in this industry is inherently complicated, we do not believe it is adequately assessed by summing the number of clinical trials and FDA filings. This is particularly true for startups whose services are not subject to FDA approval. Employed at a digital health company focused on patient navigation and virtual care, we argue much can be gained by including 3 additional components, at a minimum, when assessing clinical robustness: accreditations, externally validated methodologies, and peer-reviewed studies demonstrating impact.

First, accreditations can serve as an important proxy measure for high-quality clinical care. There are multiple well-cited frameworks for high-quality clinical care today, including the Quadruple Aim and the Institute of Medicine’s 6 aims for improvement [2,3]. Large, formally recognized accrediting bodies have used these frameworks to hold providers accountable to performing against diverse, complex care priorities. Achievements of relevant accreditations, such as the National Committee for Quality Assurance, demonstrate a commitment to a clinically robust approach to care delivery.

Second, it is not uncommon for digital health startups to rely on objective, external validation of their assumptions and results by reputable third parties. For example, a third party validated Included Health’s provider match algorithm. These studies demonstrate a startup’s commitment to a robust approach for product design and evaluation.

Finally, some digital health startups have made an investment in rigorous research that is not captured in ClinicalTrials.gov but disseminated through peer-reviewed literature. The authors acknowledged the lack of peer-reviewed literature as a study limitation. Without this component, however, well-designed studies with meaningful outcomes are overlooked. For example, we conducted a randomized controlled trial on antibiotic stewardship that found a reduction in antibiotic prescribing rates [4]. In another study, we demonstrated reductions in blood pressure among patients with elevated blood pressure who had a video visit [5]. We believe that a well-designed literature review, including terms to identify studies of interest, is a feasible and necessary addition to capture these important findings.

A clinical robustness definition that applies to all of digital health is not an easy task, and we applaud the authors for attempting this across a diverse group of startups. However, we believe that the conclusions are insufficient given the narrow definition of clinical robustness. A broader definition that varies by type of digital health company (eg, care delivery, medical technology) is critical to appropriately assess the clinical robustness of any digital health startup. 

## Abbreviations

FDA: Food and Drug Administration
